# Supplementary material for: Splice-Junction-Based Mapping of Alternative Isoforms in the Human Proteome
Source: Cell Rep. Author manuscript; Available in PMC 2020 Jan 15. (PMC6961840; doi:10.1016/j.celrep.2019.11.026)
Supplement: 2 [file NIHMS1546469-supplement-2.zip › DF1/PXD010154/Liver/Liver_17_CAMSAP3_ASPAAPADGAAPAQPSHA.pdf]

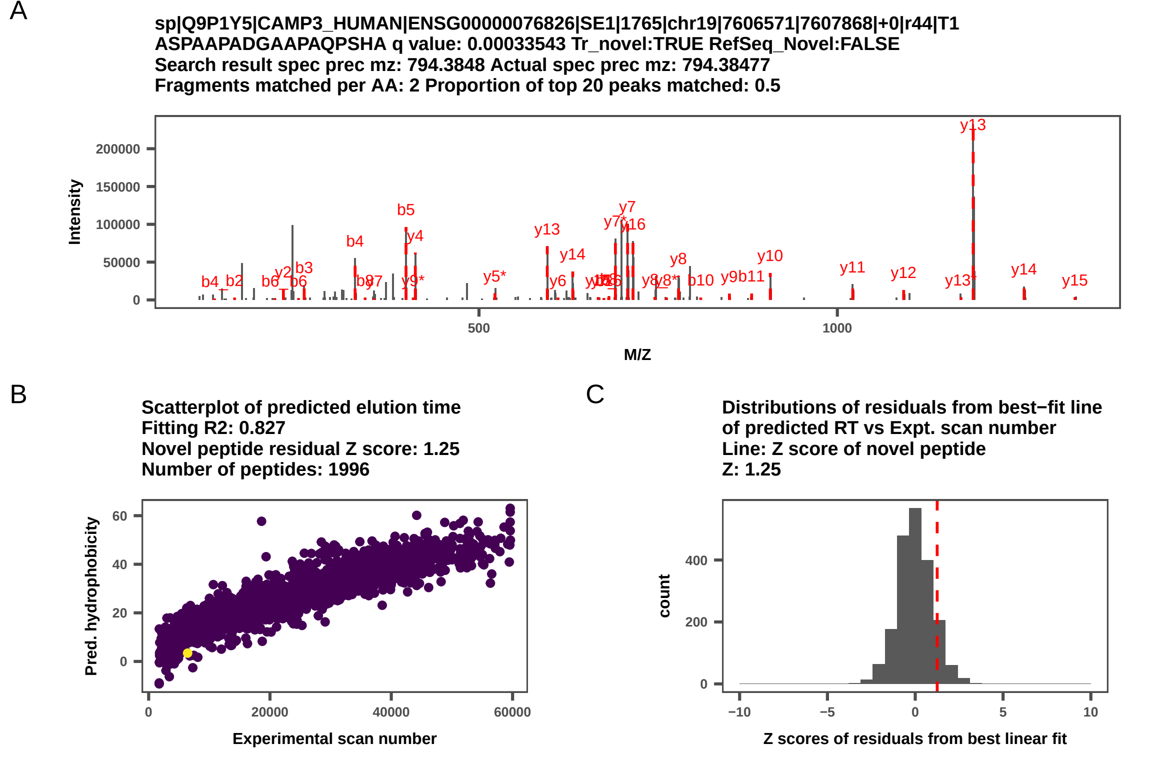

A
sp|Q9P1Y5|CAMP3_HUMAN|ENSG00000076826|SE1|1765|chr19|7606571|7607868|+0|r44|T1
ASPAAPADGAAPAQPSHA q value: 0.00033543 Tr_novel:TRUE RefSeq_Novel:FALSE
Search result spec prec mz: 794.3848 Actual spec prec mz: 794.38477
Fragments matched per AA: 2 Proportion of top 20 peaks matched: 0.5
Intensity
200000
150000
100000
50000
0
500
1000
M/Z
y13
b5
y4
b4
y7
y7*
y16
y13
y14
y8
y10
y11
y12
y13*
y14
y15
b3
y5*
y6
y9*
b10
y9b11
B
Scatterplot of predicted elution time
Fitting R2: 0.827
Novel peptide residual Z score: 1.25
Number of peptides: 1996
Pred. hydrophobicity
60
40
20
0
0
20000
40000
60000
Experimental scan number
C
Distributions of residuals from best−fit line
of predicted RT vs Expt. scan number
Line: Z score of novel peptide
Z: 1.25
count
400
200
0
−10
−5
0
5
10
Z scores of residuals from best linear fit
